# Reaching higher, landing harder: classifying individualized jump intensity zones and ground impact forces in professional male and female volleyball matches

**DOI:** 10.5114/biolsport.2026.156230

**Published:** 2025-11-24

**Authors:** André Rebelo, Irineu Loturco, Aaron T. Scanlan

**Affiliations:** 1 CIDEFES, Research Center in Sport, Physical Education, and Exercise and Health, Lusófona University, Lisbon, Portugal; 2 COD, Center of Sports Optimization, Sporting Clube de Portugal, Lisbon, Portugal; 3 NAR—Nucleus of High Performance in Sport, São Paulo, Brazil; 4 Department of Human Movement Sciences, Federal University of São Paulo, São Paulo, Brazil; 5 FSI - Football Science Institute, 18016, Granada, Spain; 6 UCAM Research Center for High Performance Sport, UCAM Universidad Católica de Murcia, Spain; 7 School of Health, Medical and Applied Sciences, Central Queensland University, Rockhampton, QLD, Australia; 8 S.P.O.R.T. Research Cluster, Central Queensland University, Rockhampton, QLD, Australia

**Keywords:** Athletic performance, Elite athletes, Jumping ability, Load, Monitoring, Team sport

## Abstract

This exploratory study aimed to (i) develop a framework to classify individualized jump intensity zones by combining relative jump height and landing impact force and (ii) explore sex- and position-based differences using these zones among volleyball players. Twenty-four players (13 male, 11 female) from two professional teams were monitored across the 2024–2025 season. Maximum jump height was determined using a spike jump test with an inertial measurement unit (IMU) for each player. In-match jumps were captured using the same IMU, with jump height and landing force recorded. Jump height was expressed as a percentage of individualized maximum jump height from the spike jump test (or in-match measurement if higher). K-means clustering defined five intensity zones for each sex. Descriptive analyses were conducted according to position (middle blockers, outside hitters, opposite hitters, and setters) and sex. Overall, 17,930 jumps in males and 14,725 jumps in females were analysed during matches. Males demonstrated significantly higher average jump heights (57.2 ± 16.3 cm vs. 41.7 ± 13.0 cm, p < 0.001, g = 1.00) and greater landing forces (10.4 ± 5.1 g vs. 8.4 ± 4.7 g, p < 0.001, g = 0.41) than females. Individualized zones were identified, ranging from very low (~25% of maximum jump height) to very high (~80% of maximum jump height) intensities. Landing impact increased progressively across zones, ranging from approximately 6 g in the lowest zone to over 12 g in the highest zone. Middle blockers and outside hitters accumulated more high- and very high-intensity jumps, while setters exhibited more moderate-intensity jumps than other positions. Males showed higher absolute and relative jump heights as well as greater landing forces than females, although intensity zone thresholds were comparable across sexes. This study introduces a novel and individualized method for classifying jump intensity in volleyball, combining jump height and landing impact forces to reflect mechanical output and stress.

## INTRODUCTION

Volleyball is a globally recognized and popular court-based team sport [1]. Although the movements performed during volleyball matchplay are varied and multi-directional, there is notable reliance on executing rapid, explosive activities—particularly vertical jumps [2]. Jumping actions are integral to serving, attacking, and blocking in volleyball, allowing players to interact with the ball above the net to influence point outcomes [3]. Jumping actions, such as those underpinning spikes and blocks, are especially important given that they contribute heavily to scoring and defensive success [3]. Consequently, jumping ability is often seen as an important performance determinant, with jumping and landing being recognised as key trainable attributes among volleyball coaches and players [4], and jump height values being used as benchmarks in player selection and training contexts within volleyball [5]. Moreover, jumping is not only frequent but also position- and sex-dependent during volleyball matchplay [6]. In this way, setters often perform more jumps, though typically at lower intensities, while outside hitters and middle blockers execute jumps less frequently but more intensely, particularly during attacks and blocks [7, 8]. Similarly, sex-based variations in spike jump performance due to strength, power, technical, and coordinative attributes [9], as well as in jump loads across training and match settings [10], have been reported in the literature. Overall, the extensive jumping demands in volleyball have led to the standard inclusion of vertical jump assessments—such as the spike and block jump tests—in performance monitoring protocols within the sport [2, 11].

The cumulative demands of jumping actions throughout volleyball match-play, particularly when repeatedly performed at high intensities, can pose significant risks to player health [12]. The high mechanical stress not only challenges the musculoskeletal system but also increases susceptibility to overuse injuries, especially when landing forces are not properly managed [13, 14]. Epidemiological data have consistently shown that most volleyball-related injuries occur at or near the net, primarily during the landing phases after blocking or attacking actions [13]. For instance, Bahr et al. [14] found that 86% of acute ankle sprains in volleyball occurred while landing, most often from blocking or attacking. From a chronic perspective, results have highlighted the relationship between high jump exposure and patellar tendinopathy, a degenerative condition of the patellar tendon linked to repetitive strain [10]. Lian et al. [15] observed that players with this condition often exhibited superior jump performance and greater body mass, suggesting that increased loading capacity may paradoxically predispose athletes to tendon degeneration. Notably, eccentric forces during landing appear to be a significant contributing factor to patellar tendinopathy, as they can be up to three times greater than concentric forces [15]. Furthermore, ground reaction forces during landings from jumps can reach around twice that of body weight forces among volleyball players [16, 17]. Consequently, landing forces associated with jumps are important to consider, because when repeated across long competitive seasons and without sufficient recovery or neuromuscular control, these forces can exacerbate acute and chronic injury risk [10, 17].

In this context, understanding the intensity of jump outputs (e.g., height) and landings (e.g., impact force) seems logically essential. However, while jump output metrics have been widely studied as markers of readiness and performance in volleyball players [18–20], no known studies have simultaneously assessed landing forces and their relation to jump height across varied intensities in volleyball match-play. In fact, separate reviews of the literature show that studies in this area predominantly measure jump height and/or jump count when monitoring player loads in volleyball contexts [6, 21, 22]. Consequently, dual analysis of jump height alongside landing force is scarce in the volleyball literature but is needed to build load profiles that reflect not just how often and how high players jump, but how intensely they land from each jump. When quantifying such demands in high-performance sport settings, it is crucial that an individualized approach is adopted to ensure principles of individuality and specificity are followed for optimal translation of outcomes to practice. In this regard, player-specific thresholds set as proportions of maximal physical capacities or outputs are customarily used to delineate intensity zones when developing individualized load monitoring approaches in team sports [23–25].

Evidence from other team sports suggests that individualized intensity zones produce different activity profiles compared to zones based on arbitrary thresholds, more accurately reflecting the actual demands placed on each player [25], as well as the associated injury risks [26]. However, recent systematic evidence on the use of individualized thresholds to derive intensity zones for load monitoring in team sports highlights the lack of research attention dedicated to volleyball on this topic [23], with no known wider literature applying this concept specifically to jump metrics. Translating this concept to volleyball, using individualized zones based on percentages of maximum jump height instead of absolute, arbitrary thresholds has been called for in the literature [22] and may allow for more nuanced load monitoring by accounting for the wide variability in physical capacities across players and positions. This individualization is particularly important for key applications of load monitoring data, such as guiding training load management, optimizing performance, and minimizing injury risk [27].

Given the absence of intensity zones based on individualized thresholds to quantify load in volleyball—and the established use of such an approach with jump metrics in team sports—this exploratory study aimed to establish a data-driven method for defining these zones based on two key external load variables: relative jump height and landing impact force. Using match-derived data collected throughout a full competitive season, we sought to identify jump intensity zones with individualized thresholds in professional volleyball players—separately for males and females—and to quantify jump outcomes by position using this approach. We hypothesized that: (i) a five-zone clustering approach would clearly delineate jump intensities in an individualized manner, based on the percentage of maximal height and landing impact force; (ii) male and female players would exhibit distinct intensity zone thresholds, reflecting differences in performance capacities and mechanical loading; and (iii) the distribution of jumps across these zones would vary by playing position.

## MATERIALS AND METHODS

### Study Design

The development of this manuscript adhered to the Strengthening the Reporting of Observational Studies in Epidemiology (STROBE) guidelines for cohort studies [28], and the completed STROBE checklist, including page references for each item is provided as a Supplementary Table 1. This exploratory, observational, retrospective cohort study analysed all official competitive matches played by two professional indoor volleyball teams—one male and one female—across a full competitive season. Data were collected during the 2024–2025 season, spanning from September 2024 to May 2025, and included 43 matches for the male team and 35 matches for the female team. All official matches were considered, including domestic league and cup competitions, Supercup fixtures, and European club tournaments. No experimental interventions or modifications to training or match routines were introduced, ensuring that the study setting reflected the natural, high-performance environment in which both teams competed throughout the season. The study methods were reviewed and approved by the Lusófona University Human Research Ethics Committee (approval number J0425). The experimental protocol conformed to the principles of the Declaration of Helsinki, established by the World Medical Association for research involving human participants [29]. Additionally, all procedures adhered to recognised ethical standards for sports medicine research [30].

### Participants

A total of 24 volleyball players participated in this study, comprising 13 males and 11 females. All players met the criteria for Tier 4 athletes within the Participant Classification Framework [31].

At the start of data collection, male players had a mean age of 29.8 ± 5.5 years, body mass of 95.0 ± 7.6 kg, and height of 196.0 ± 7.3 cm. Their positional distribution included four middle blockers, five outside hitters, two opposite hitters, and two setters. Female players had a mean age of 27.0 ± 5.0 years, body mass of 76.4 ± 9.4 kg, and height of 181.5 ± 4.9 cm. Their positional distribution included four middle blockers, three outside hitters, two opposite hitters, and two setters. Players occupying the libero position were excluded from the analysis due to their limited engagement in jumping actions during match-play, which could confound the determination of jump-based intensity zones across the remaining positions. For the positional analysis, players who averaged fewer than 15 jumps per set in a given match were excluded, as previous research has shown that volleyball players typically perform more than 15 jumps per set [2]. This criterion helped eliminate cases of limited match involvement, such as tactical substitutions for single rotations (e.g., serving or blocking only).

### Variables and Data Collection

Each player’s maximum jump height was established using a spike jump test conducted prior to the competitive season. This test was selected due to its high specificity to jumping actions performed during volleyball match-play. Previous research in volleyball supports the use of movement-specific jump assessments, including the spike jump test, particularly when measured with sport-relevant tools such as the Vertec device [2, 11].

Players performed maximal vertical spike jumps using a Vertec apparatus (Sports Imports, Hilliard, OH, USA), a widely used tool for assessing vertical jump height in volleyball players [11]. The Vertec consists of a vertical pole with horizontal vanes that are displaced upon contact with the fingers, allowing for accurate measurement of jump-and-reach performance—highly relevant to in-match actions [32]. Prior to testing, each player completed a standardized warm-up consisting of 5 minutes of cycling on a stationary cycle ergometer at a self-selected low intensity, followed by dynamic mobility and movement preparation drills, including multidirectional skips, lunges with trunk rotation, and submaximal plyometric exercises. This sequence was followed by two submaximal jump trials and one near-maximal jump trial to ensure adequate preparation for the test performance.

The test protocol involved a three-step approach and a one-handed reach, replicating the technique typically used in spike actions in match-play [33]. When performed using the Vertec apparatus, the spike jump test has demonstrated moderate reliability in volleyball players (intraclass correlation coefficient [ICC] = 0.63–0.75; standard error of measurement = 1.56–1.61 cm) [34]. Players completed three maximal trials, with 3 minutes of passive recovery between attempts. During each jump, athletes wore an inertial measurement unit (IMU)—the VERT Classic device (MyVert, Fort Lauderdale, FL, USA)—secured in a belt positioned at the iliac crest. This device includes a tri-axial accelerometer, gyroscope, and magnetometer, and transmitted data in real-time via Bluetooth to an iPad app (VERT Team System, Mayfonk Athletic, LLC). The VERT has been validated for jump height measurement against a reference laboratory-based motion analysis system, as well as for jump detection during match-play when compared to video analysis in volleyball players [35]. The VERT was used to determine the actual jump height (cm) for each spike jump attempt, and the highest value among the three maximal trials was retained as the reference for calculating the percentage of maximum jump height for each jump detected during match-play. If, at any point during the season, a player exceeded their initial maximum value during a match, their reference was prospectively updated from that point onward. This approach ensured that the calculated percentages remained reflective of each player’s evolving capacity—an update that occurred on four occasions across four different players in this study.

During all official matches across the 2024–2025 competitive season, players wore the same IMU described previously—the VERT Classic—positioned at the level of the iliac crest using a secured belt. Each player was assigned the same device for the entire season to eliminate the risk of inter-device variability. The device captured jump height (cm) and landing impact force (G-force) for each jump performed in real-time via the connected iPad app. Raw data for absolute jump height and landing force were extracted from the VERT cloud platform (VERT Sports, Fort Lauderdale, FL, USA). Relative jump height (expressed as a percentage of each player’s individualized maximum, as determined during the spike jump test or detected during match-play) was subsequently calculated manually in a separate spreadsheet. Only valid jumps performed during active match-play were retained. Non-active periods—including warm-ups, timeouts, substitutions, or time spent on the bench—were excluded in real-time by placing players in “pause” mode using the VERT app. Identical data collection and processing procedures were applied to both male and female teams to ensure consistency and comparability.

### Statistical Analysis

All statistical analyses were conducted using IBM SPSS Statistics (version 27, IBM Corp., Armonk, NY, USA). Descriptive statistics are presented as means ± standard deviations, along with minimum and maximum values where relevant.

To define individualized jump intensity zones, k-means clustering was performed separately for male and female players using all valid in-game jumps recorded across the competitive season. The clustering model simultaneously incorporated two variables for each jump: relative jump height (expressed as a percentage of each player’s maximum) and the corresponding landing impact force. This approach ensured that each cluster captured the combined intensity of jump output and its associated landing load. The number of clusters was set to five, corresponding to a practical classification of jump efforts: very low, low, moderate, high, and very high intensity. This decision was supported by both methodological and applied considerations. First, five-zone frameworks are commonly used in team sport literature for individualized external load monitoring (e.g., individualized running speed zones) [36]. Second, a recent review [23] highlighted that most studies reporting individualized thresholds adopt similar five-tier classifications (e.g., “very high” to “very low”). Third, five zones provide a balance between interpretability and granularity, facilitating practical application in sport settings. Following clustering, total jump counts per player within each zone were calculated. Descriptive statistics for jump distribution were then computed by position × zone combination to support positional demand comparisons.

To compare jump patterns between male and female players, player-level averages were calculated for two primary match-play variables: relative jump height and landing impact force. Specifically, the mean of all valid jumps performed during match-play was computed for each player, generating one value per player per variable. These player-level values were then used to calculate group means and standard deviations by sex, ensuring equal weighting of individuals regardless of jump frequency. Between-sex comparisons were assessed using independent samples t-tests, with Levene’s test applied to evaluate the assumption of homogeneity of variances. In cases of unequal variances, Welch’s t-test was used. Effect sizes were computed using Hedges’ *g*, which adjusts for differences in group sample sizes. Statistical significance was set at *p* < 0.05. For positional analyses, total jumps were grouped according to player role (middle blocker, outside hitter, opposite hitter, and setter) and summarized by jump intensity zone.

## RESULTS

Table 1 presents the descriptive statistics for jump performance metrics in the male and female volleyball players included in analyses. Males demonstrated significantly higher relative jump height (mean difference = 4.02%, *p* < 0.001, *g* = 0.24), as well as significantly greater average jump height (mean difference = 15.50 cm, *p* < 0.001, *g* = 1.00) and landing impact force (mean difference = 2.00 g, *p* < 0.001, *g* = 0.41) during matches compared to female players.

**TABLE 1:** Descriptive data (presented as mean ± standard deviation where relevant) for jump metrics in professional male and female volleyball players.

Player sex	Number of players	Maximum jump height (cm)	Total matches	Average jump height (cm)	Percentage of maximum jump height (%)	Landing impact force (G-force)
Male	13	92.5 ± 7.0	43	57.2 ± 16.3	61.8 ± 17.3	10.4 ± 5.1
Female	11	70.4 ± 7.6	35	41.7 ± 13.0	57.8 ± 16.6	8.4 ± 4.7

**FIG. 1:**
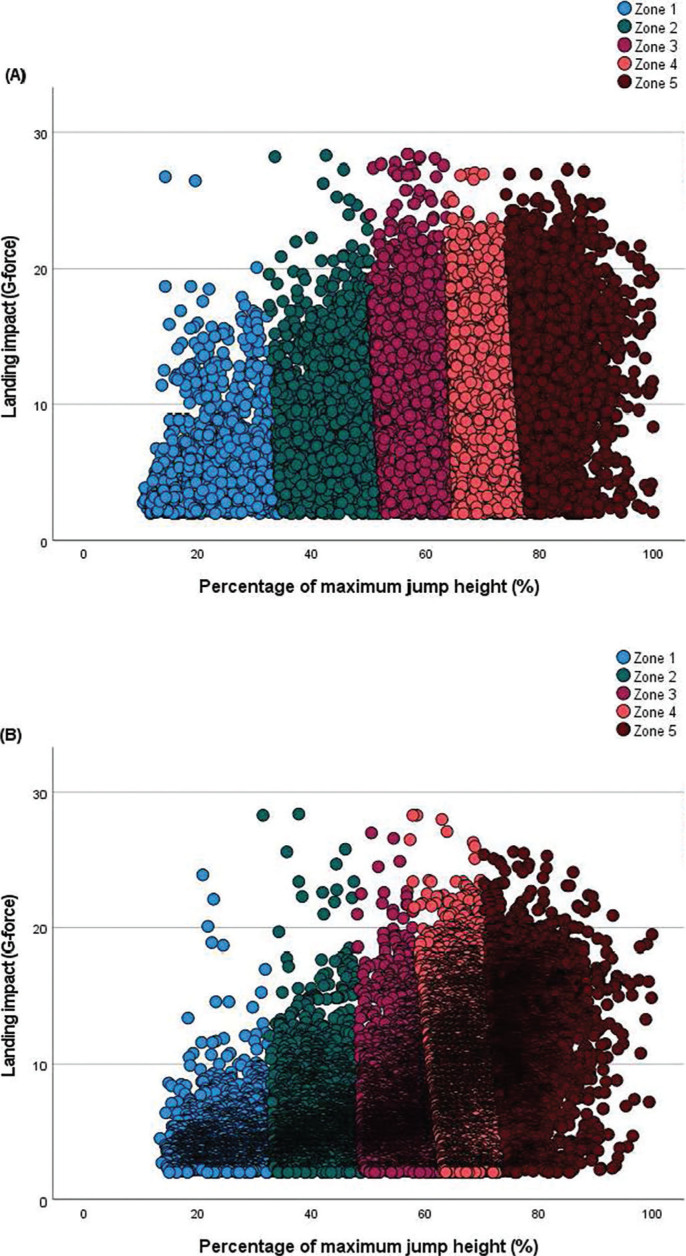
Relationship between landing impact force and relative jump height across the five individualized intensity zones during match-play. Scatterplots of landing impact force (G-force) according to jump intensity (jump height expressed as a percentage of each player’s maximum jump height) during professional volleyball matches: (A) male and (B) female players. Coloured markers indicate the five individualized intensity zones identified through k-means cluster analysis: Zone 1 (very low), Zone 2 (low), Zone 3 (moderate), Zone 4 (high), and Zone 5 (very high).

The five distinct jump intensity zones—based on the percentage of maximum jump height and the associated landing forces—determined separately for males and females are shown in Table 2. Intensity zone thresholds varied only slightly between sexes, with both male and female players exhibiting a progressive increase in relative jump height and corresponding landing forces across zones as intensity increased. The clear separation between jump intensity zones is illustrated in Figure 1 for male and female players.

**TABLE 2:** Final cluster centers from k-means cluster analyses for individualized jump intensity zones in professional male and female volleyball players.

Player sex	Zone	Percentage of maximum jump height (%)	Landing impact force (G-force)	Interpretation
Male	1	23.3	5.3	Very low intensity
	2	44.5	8.4	Low intensity
	3	58.9	10.4	Moderate intensity
	4	70.5	11.2	High intensity
	5	81.8	13.1	Very high intensity

Female	1	24.9	4.5	Very low intensity
	2	41.3	6.0	Low intensity
	3	56.0	6.9	Moderate intensity
	4	67.8	9.9	High intensity
	5	78.2	12.5	Very high intensity

A total of 17,930 jumps were recorded for male players and 14,725 jumps for female players during match-play (Table 3). In both sexes, the number of jumps generally increased from Zone 1 (very low intensity) to Zone 4 (high intensity), followed by a decrease in Zone 5 (very high intensity).

**TABLE 3:** Distribution of jumps across individualized intensity zones with corresponding jump height and landing impact force values (presented as mean ± standard deviation) in professional male and female volleyball players.

Player sex	Zone	Number of jumps	Percentage of maximum jump height (%)	Landing impact force (G-force)
Male	1	1551	23.3 ± 6.1	5.3 ± 3.2
	2	2638	44.5 ± 5.0	8.4 ± 4.3
	3	4833	58.9 ± 3.7	10.4 ± 4.7
	4	5152	70.5 ± 3.4	11.2 ± 4.8
	5	3756	81.8 ± 4.6	13.1 ± 5.1

Female	1	1606	24.9 ± 5.1	4.5 ± 2.1
	2	2184	41.3 ± 4.5	6.0 ± 3.3
	3	3532	56.0 ± 3.8	6.9 ± 3.5
	4	4880	66.8 ± 3.2	9.9 ± 4.5
	5	2523	78.2 ± 4.9	12.3 ± 4.7

**FIG. 2:**
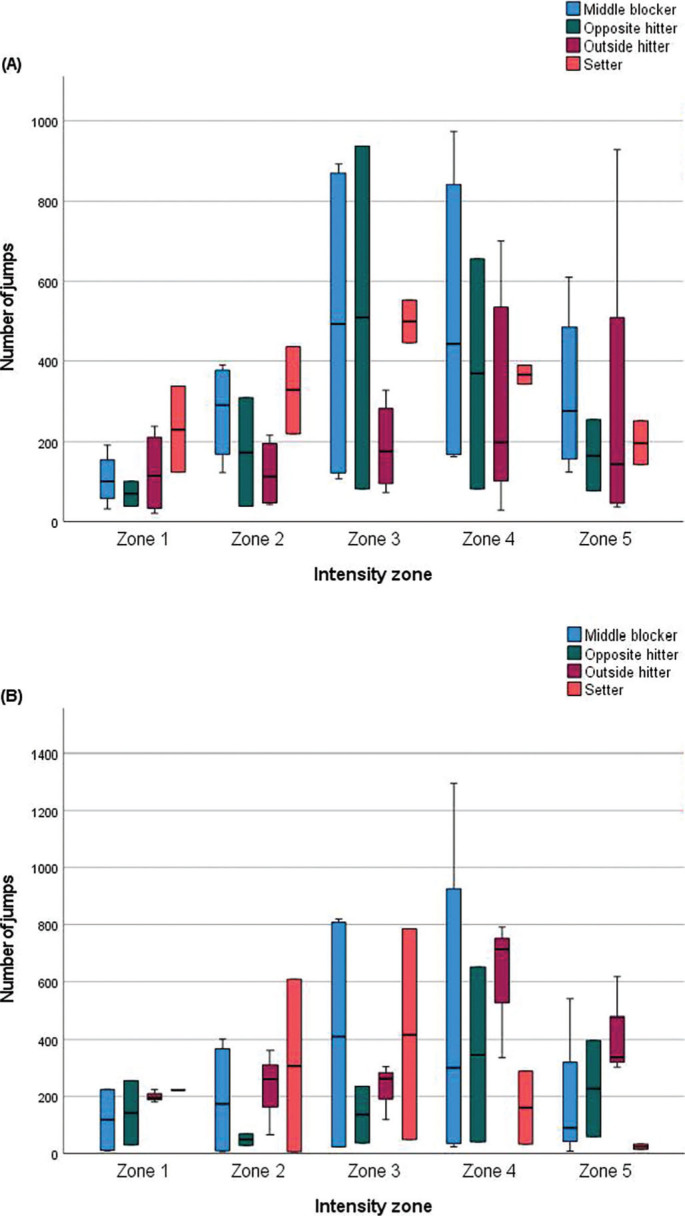
Positional distribution of jump counts across individualized intensity zones in professional volleyball. Boxplots showing the distribution of jumps across the five individualized intensity zones according to playing position in professional (A) male and (B) female volleyball players. The central line within each box represents the mean, the edges represent the standard deviation, and the whiskers indicate the range.

Figure 2A illustrates the distribution of jumps per player across the five individualized intensity zones according to playing position in the male team. Setters showed a clear peak in jump counts within Zone 3 (500 ± 76; range: 446–553), along with a high number of jumps in Zone 4 (366 ± 33; range: 342–389) and a moderate count in Zone 5 (197 ± 76; range: 143–250). They also accumulated substantial jump counts in the lower-intensity zones: Zone 1 (231 ± 151; range: 124–337) and Zone 2 (328 ± 153; range: 220–436). Opposite hitters recorded their highest jump counts in Zone 3 (509 ± 604; range: 82–936) and Zone 4 (369 ± 406; range: 82–656), with a notable number of jumps also observed in Zone 5 (165 ± 125; range: 77–253). Lower jump counts were observed in Zone 1 (70 ± 44; range: 39–101) and Zone 2 (174 ± 190; range: 39–308) among opposite hitters. Middle blockers exhibited their highest jump counts in Zone 4 (506 ± 404; range: 163–973), followed closely by Zone 3 (497 ± 433; range: 107–894) and Zone 5 (321 ± 217; range: 124–610). Jump counts in the lower intensity zones were also considerable, with 114 ± 80 jumps (range: 32–192) in Zone 1 and 273 ± 126 jumps (range: 123–390) in Zone 2. Outside hitters demonstrated a more progressive distribution pattern, with increasing jump counts from Zone 1 to Zone 5: Zone 1 (115 ± 93, range: 21–239); Zone 2 (122 ± 88, range 43–217); Zone 3 (188 ± 115, range: 73–327); Zone 4 (313 ± 291, range: 29–701); and Zone 5 (333 ± 384, range: 37–927).

In the female team, distinct positional patterns were observed in the distribution of jumps across the five individualized intensity zones (Figure 2B). Setters recorded their highest jump counts in Zone 3 (417 ± 520; range: 49–785) and Zone 2 (307 ± 426; range: 6–608), with a progressive decline across higher intensity zones, culminating in a markedly lower count in Zone 5 (24 ± 13; range: 15–33). Opposite hitters exhibited a bimodal distribution, with substantial jump counts in Zone 4 (346 ± 431; range: 41–651) and Zone 5 (228 ± 239; range: 59–397), alongside moderate counts in Zone 1 (143 ± 160; range: 30–256) and Zone 3 (137 ± 141; range: 37–236). Middle blockers showed the lowest jump counts in the lower-intensity zones: Zone 1 (118 ± 122, range: 9–226); and Zone 2 (189 ± 209, range: 6–401), with the highest jump counts observed in Zone 3 (416 ± 453; range: 23–819) and Zone 4 (481 ± 596; range: 24–1296). Outside hitters demonstrated the highest jump counts in the most intense zones, peaking in Zone 4 (614 ± 242; range: 337–791) and accumulating substantial activity in Zone 5 (419 ± 172.1; range: 303–617). Their jump counts decreased progressively across the lower intensity zones, with 229 ± 98 in Zone 3 (range: 119–305) and 201 ± 22 in Zone 1 (range: 181–225).

## DISCUSSION

This study aimed to define individualized jump intensity zones and the accompanying landing forces in professional volleyball players using a data-driven clustering approach based on relative jump height. Across a full competitive season, more than 32,000 jumps were analysed from male and female matches. The application of k-means clustering successfully identified five distinct intensity zones for both sexes, reflecting progressive increases in jump height as a percentage of individualized maximum values and in landing forces. As hypothesized, the distribution of jumps across zones varied by playing position. Additionally, male players exhibited higher absolute and relative jump heights, as well as greater landing forces, compared to female players—supporting the second hypothesis regarding sexbased differences.

As expected, the results of this study showed concomitant increases in relative jump height and landing force as intensity increased across the five zones. This finding confirms that as players jump closer to their maximum height, the mechanical demands during landing exhibit parallel increases during match-play. These results align with prior biomechanical studies reporting greater ground reaction forces and impact accelerations as jump height increases [37]. For instance, ground reaction forces during landings can reach approximately twice that of body weight among volleyball players [16, 17], reinforcing the link between jump height and landing load. This relationship also validates the decision to simultaneously incorporate both relative jump height and landing impact force into the clustering model, allowing each jump to be classified based on its combined mechanical output during take-off and landing.

While jump height has traditionally been used in volleyball to assess performance or track player loads [6, 19, 20], landing forces are rarely quantified in match scenarios [6], despite their direct relevance to injury risk [38]. Including both variables enabled the identification of intensity zones that reflect not only the output of a jump, but also the mechanical stress of landing—an aspect often overlooked in jump monitoring. Considering landing forces is particularly important in a sport like volleyball, where repeated high-load landings can contribute to overuse injuries, especially in tendinous structures [10, 15]. Thus, dual-variable clustering allows a more individualized and accurate representation of mechanical load, holding potential relevance for both performance optimization and injury prevention strategies in practice.

The present study revealed clear sex-based differences in both jump height and landing force across all jump intensity zones, with males demonstrating higher absolute and relative jump heights, as well as greater landing forces. These results are consistent with previous findings in volleyball, where male players consistently outperformed female players in vertical jump assessments, likely due to higher levels of muscle mass and lower-body strength [2]. Interestingly, despite these absolute differences, the clustering approach yielded relatively similar zone thresholds when jump height was expressed as a percentage of each player’s maximum jump height. This finding suggests that while male and female players may generate different absolute mechanical outputs, their relative intensity profiles as a function of their own capacity are more comparable. This is an important finding, as it supports the use of individualized zones when interpreting sex-specific jump load data in volleyball settings, allowing for a more precise and meaningful interpretation of the demands imposed on players.

The higher landing forces observed in males are also likely multifactorial, with their greater jump height likely increasing landing impact forces [39], alongside differences in landing technique, body mass, and neuromuscular control potentially contributing as well [40]. Bressel and Cronin [38] observed that female players tend to adopt a softer landing strategy, characterized by increased knee and hip flexion and longer landing time, which may help attenuate peak forces. In contrast, male players often use stiffer landing mechanics, generating higher impact loads despite similar or even lower relative jump intensities [38]. These neuromechanical differences help explain the sex-based disparities in landing forces observed in the present study and are supported by prior research highlighting sex-specific adaptations during high-impact activities [39, 41].

The distribution of jumps across intensity zones varied substantially by playing positions, reflecting the distinct technical and tactical demands placed on players within each role. Among male players, middle blockers accumulated the highest number of jumps at higher intensities (Zones 4 and 5), likely due to their role in performing successive explosive actions such as blocking and first-tempo attacks, often with limited recovery time between efforts [42]. These findings align with those reported in a systematic review, which showed that middle blockers engage in the highest number of jumps per match and experience some of the greatest lower-limb demands during match-play [6]. In contrast, among female players, outside hitters recorded the greatest volume of high-intensity jumps—a pattern likely reflecting their match responsibilities in terminating rallies and attacking from both the front and back rows, requiring powerful approaches and spikes from varied court positions [43]. The elevated frequency of high-intensity jumps among outside hitters also aligns with their involvement in offensive and defensive transitions, increasing their cumulative load throughout matches. Setters, in contrast, displayed a more balanced distribution of jumps across intensity zones in both sexes, with the highest occurring in Zone 3. This pattern may be explained by the functional nature of the jumping tasks performed by setters, which includes block participation and jump setting—typically performed at submaximal intensities and without full-speed approaches [44]. Opposite hitters presented a rather heterogeneous jump profile across intensity zones, with elevated jump counts at moderate and high intensities, particularly among males. This finding for opposite hitters is likely reflective of their dual offensive and blocking responsibilities during match-play, often positioned against the strongest outside hitter on the opposing team and receiving a high volume of sets during offensive phases [45]. Taken together, these position-specific findings reinforce the relevance of an individualized approach to load profiling to better contextualize jump demands within technical-tactical roles among volleyball players.

Despite the novel load monitoring outcomes provided in volleyball settings, notable limitations should be considered when interpreting our findings. First, although the dataset was extensive in terms of the overall number of jumps recorded during matches, the sample was restricted to two professional teams. As such, the generalizability of the results to other teams and competitions may be limited, particularly if different playing styles, physical profiles, or tactical systems are present. Moreover, this limitation especially restricted the position-specific analyses—and sex-specific analyses to a lesser extent—given the smaller number of players within each group when categorized in this way. For this reason, the present study should be interpreted as exploratory, aiming to generate initial insights into individualized jump intensity zones and positional demands in volleyball. Second, the k-means clustering analysis was conducted across all players within each sex as an initial exploration of an individualized approach to jump load monitoring. In this regard, the data could be further individualized by performing k-means cluster analyses at an individual player level, which should be addressed in future research. This innovative approach would allow for further individualization of jump demands based on each player’s unique jumplanding profile and may uncover patterns in outcomes that are masked by group-level analyses. Moreover, although we simultaneously clustered relative jump height and landing impact forces per event, we did not explore intra-individual variability in how these two variables relate to one another. Future research should examine the extent to which players with similar relative jump outputs exhibit different landing forces—potentially due to differences in body mass, neuromuscular control, or landing technique—as this would offer an additional layer of individualization and may enhance both performance profiling and injury risk screening. Third, while the five-zone structure adopted in this study aligns with frameworks commonly used in individualized external load monitoring in other sports [23], the selection of five clusters remains somewhat arbitrary. It is possible that a different number of zones (e.g., three or four) could yield different insights or provide greater utility depending on the context or desired sensitivity of monitoring. Fourth, our study encompassed only external load monitoring—specifically jump demands—excluding other relevant external load metrics associated with explosive actions in volleyball (e.g., accelerations, decelerations, changes of direction, running) as well as internal load metrics. Future studies could expand on our work by exploring the application of individualized load monitoring using k-means cluster analyses to derive intensities for other external load metrics of interest. Moreover, such research should aim to integrate internal load markers, such as heart rate, session rating of perceived exertion, or hormonal responses, to examine how individualized external demands correspond to the physiological and perceptual stress experienced. Finally, our study encompassed a single season; longitudinal research tracking changes in jump demands using individualized intensity zones over multiple seasons could help identify trends related to fatigue, adaptation, or injury risk, considering contextual factors such as training phase [46] or congested fixture schedules [47].

### Practical Applications

The adoption of individualized intensity zones to quantify jump load based on volleyball players’ maximum jump height offers a more precise and context-sensitive method for monitoring external load during match-play. In this study, we defined five distinct jump intensity zones for each sex using k-means clustering. To enhance practical application, we propose rounded percentage thresholds that approximate the original cluster centers, acknowledging slight sexbased differences. These thresholds, expressed as a percentage of each player’s highest jumps, are: Zone 1 (very low intensity) = < 45%; Zone 2 (low intensity) = 45–59%; Zone 3 (moderate intensity) = 60–69%; Zone 4 (high intensity) = 70–79%; and Zone 5 (very high intensity) = ≥ 80%. Although landing forces varied slightly between sexes, they increased progressively with jump intensity, supporting the ecological validity of this classification. Overall, individualized jump zones provide superior contextualization compared to traditional absolute thresholds (e.g., jumps > 40 cm), which did not account for differences in physical capacities, positions, or sex. For example, a 45-cm jump could be submaximal for one player but near-maximal for another. Using percentage-based thresholds tailored to each player’s capacity allows for more accurate interpretation of match demands and helps reduce misleading conclusions. This individualized strategy has important implications for load management, performance optimization, and injury risk reduction, especially given the potential fatigue and tissue strain associated with repeated highintensity landings [10]. It may also help guide return-to-play protocols, allowing injured players to progressively increase exposure to higher intensity zones in a controlled manner. Furthermore, integrating these zones into real-time monitoring systems can help flag players accumulating unusually high volumes at high intensities, prompting timely training adjustments and recovery interventions. Longitudinal tracking of exposure to each intensity zone throughout the season also offers a valuable tool for chronic load surveillance, helping coaches identify trends in load adaptation, performance plateaus, or spikes that could signal increased injury risk. In summary, this zone-based framework offers a more individualized and evidence-informed model for jump load monitoring in volleyball, providing practitioners with a solid foundation for designing more precise and effective training programs.

## CONCLUSIONS

This study provides a novel framework for defining individualized jump intensity zones in professional volleyball players, using extensive in-game monitoring data collected over an entire competitive season. By combining relative jump height and associated landing forces into a five-zone model, we captured both the performance output and mechanical stress of jumping actions in a sex-specific manner. Across 32,655 in-game jumps (17,930 in males and 14,725 in females), landing forces increased progressively with jump intensity, ranging from approximately 6 g in low-intensity actions to over 12 g in the highest intensity zone. The results revealed clear differences in jump profiles between playing positions and sexes; male players exhibited higher average jump heights (+15.5 cm) and landing forces (+2.0 g) compared to females, although k-means cluster analyses showed that similar relative thresholds could be applied across males and females when expressed as a percentage of maximum jump height. This individualized approach offers practitioners a more precise and context-specific tool for monitoring player jump demands, with practical applications in load management, injury prevention, and returnto-play protocols. Future research should refine this model through individual-level profiling and integration with internal load markers to further optimize player care in professional volleyball settings.

## Supplementary Material

Reaching higher, landing harder: classifying individualized jump intensity zones and ground impact forces in professional male and female volleyball matches
